# Prévalence du tabagisme chez le personnel de l'Hôpital Général de Douala, Cameroun

**Published:** 2012-02-16

**Authors:** Bertrand Hugo Mbatchou Ngahane, Henry Luma, Mor Ndiaye, Yacouba Mapoure Njankouo, Salomon Mbahe, Adeline Wandji, Elvis Temfack, Albert Mouelle Sone, Bertrand Dautzenberg

**Affiliations:** 1 Service de Médecine Interne, Hôpital Général de Douala-Cameroun; 2 Service de Médecine du Travail, Université Cheikh Anta Diop, Dakar-Sénégal; 3 Service de Pneumologie, Hôpital Laquintinie de Douala-Cameroun; 4 Service de Pneumologie, Hôpital Pitié Salpêtrière, Paris-France

**Keywords:** Tabagisme, Afrique, épidémiologie, hôpital, Cameroun

## Abstract

**Introduction:**

La prévalence du tabagisme parmi le personnel de santé hospitalier au Cameroun n'est pas connue alors que le tabagisme est en expansion dans ce pays avec 13,2% de fumeurs selon l'OMS. Pour combler ce manque une enquête sur les consommations, les connaissances, opinions et attitudes vis-à-vis des fumeurs a été conduite à l'Hôpital Général de Douala, l'un des hôpitaux de référence du Cameroun.

**Méthodes:**

Du 1er au 30 Avril 2010, des questionnaires anonymes ont été distribués par des enquêteurs dans les services ou via les surveillants et recueillis et analysés de façon anonyme.

**Résultats:**

Sur 402 questionnaires distribués 277 ont été récupérés. La prévalence de fumeurs est de 3,6% parmi les soignants et de 9,4% parmi les autres personnels soit en moyenne sur l'ensemble de l'hôpital 5,4%. Les produits fumés étaient toujours des cigarettes. L'initiation du tabagisme à souvent été tardive (21,5 ans) et la dépendance est absente ou faible chez 33% des fumeurs. Les personnes pensent que c'est leur devoir de questionner sur le tabac et de prendre en charge les fumeurs, mais ils sont presque un sur deux à ignorer la loi Camerounaise.

**Conclusion:**

Le tabagisme chez le personnel hospitalier est une réalité au Cameroun; le personnel soignant et les pouvoirs publics devraient s'impliquer davantage dans la lutte contre ce fléau qui est en expansion dans les pays du sud.

## Introduction

Le tabagisme constitue un enjeu majeur en termes de santé publique dans le monde entier, en raison de la mortalité et de la morbidité qui lui sont liées. Il constitue la première cause de décès évitable dans le monde. Il est responsable de 5 millions de décès chaque année; la plupart de ces décès sont observés dans les pays de faible revenu. Si la tendance actuelle persiste, on observera 8 millions de décès annuel d'ici 2030 [1].

Au Cameroun, pays de 19,5 millions d'habitants, dont plus de la moitié en dessous de 18 ans, l'Organisation Mondiale de la Santé estime la prévalence du tabagisme à 13,2% [1]. Une enquête récente réalisée en milieu scolaire chez les adolescents de 13 à 15 ans estimait à 13,4% la prévalence des usagers de tout produit de tabac, tandis que la prévalence des fumeurs de cigarette était de 5,7% [2]. Le tabagisme chez le personnel de santé est une réalité comme le démontrent plusieurs auteurs africains [3–6]. Au Cameroun, il n'existe pas de données sur le tabagisme chez le personnel de santé.

Dans ce travail, nous nous proposons de déterminer la prévalence du tabagisme parmi le personnel de l'Hôpital Général de Douala et de décrire les attitudes face au tabagisme.

## Méthodes

### Cadre de l’étude

Il s'agit d'une étude prospective et transversale qui s'est déroulée à l'Hôpital Général de Douala du 1er au 30 avril 2010. Elle a été conduite par un médecin de l'hôpital. Cet hôpital est un centre hospitalier de référence au Cameroun et dans la sous-région Afrique Centrale. Il comporte plusieurs services: urgence, réanimation, médecine interne, chirurgie, gynécologie obstétrique, pédiatrie, ORL, ophtalmologie, chirurgie dentaire, imagerie médicale, oncologie, biologie, laboratoire et les services administratif et technique.

### Population d’étude

L’étude concernait le personnel soignant et non soignant de l'Hôpital Général de Douala regroupé en 4 catégories : 1) les médecins; auxquels ont été associés les pharmaciens; 2) le personnel paramédical qui comportait les infirmiers de soins, les laborantins, les techniciens de radiologie, les sages-femmes, les kinésithérapeutes; 3) les agents de service hospitaliers non soignants (agents, agents temporaires, agents de maintenance, personnel de cuisine, de buanderie, de lingerie); 4) le personnel administratif (secrétaires médicaux et d'administration, économes, personnel des affaires professionnelles, d'hygiène et de comptabilité, ingénieurs, techniciens …)

### Recueil des données et analyse statistique

Nous avons élaboré un questionnaire anonyme standardisé en français. Etaient répertoriés dans ce questionnaire les caractéristiques sociodémographiques du personnel, le statut tabagique et la dépendance, les connaissances de la réglementation et les attitudes vis-à-vis des fumeurs.

Etait considéré comme fumeur celui qui déclare fumer régulièrement ou occasionnellement. L'ex-fumeur était celui qui déclare avoir arrêté de fumer depuis au moins 6 mois; les autres étaient considérés comme des non-fumeurs.

Après avoir obtenu l'autorisation de la direction de l'hôpital, le questionnaire a été distribué soit individuellement, soit par l'intermédiaire des surveillants dans les différents services. Le questionnaire rempli était immédiatement rendu ou retourné plus tard selon la disponibilité des répondants. L'enquête était faite auprès du personnel présent dans le service au moment de notre passage. Certains questionnaires ne nous ont pas été retournés.

Les données ont été saisies et analysées à l'aide du logiciel SPSS 11.5 pour Windows.

## Résultats

Nous avons distribué 402 questionnaires aux personnels des services lors du passage des enquêteurs. Au total 277 des 402 questionnaires ont été retournés, soit un taux de réponse de 68,9%.

## Caractéristiques de la population d’étude

La population était constituée de 51,6% de femmes et de 48,4% d'hommes. Le personnel paramédical (n=145) était la catégorie professionnelle la plus représentée (52,3%) (Tableau 1).

**Tableau 1:** Répartition de la population des personnels de l'hôpital de Douala selon la catégorie professionnelle

Catégorie professionnelle	Effectif	Pourcentage
Médecin	47	17%
Paramédical	145	52,3%
Administratif	54	19,5%
Technique	31	11,2%

L’âge moyen de notre population était de 41,8 ± 9,1 ans, avec des extrêmes de 23 et de 61 ans. Cette moyenne d’âge était de 44,6±8,9 ans chez les hommes et de 39,2 ± 8,4 ans chez la femme. La tranche d’âge la plus représentée était celle de 41 à 50 ans (38,4%). L'ancienneté dans la profession était en moyenne de 14,2 ± 8,8 avec des extrêmes de 25 jours à 37 ans.

Les fumeurs (n = 15) représentaient 5,4% de notre population d’étude; les ex-fumeurs 13,4% et les non fumeurs 81,2%. La prévalence du tabagisme chez le personnel soignant était de 3,6% contre 9,4% chez le personnel hospitalier non soignant.

### Caractéristiques des fumeurs

**Figure 1:**
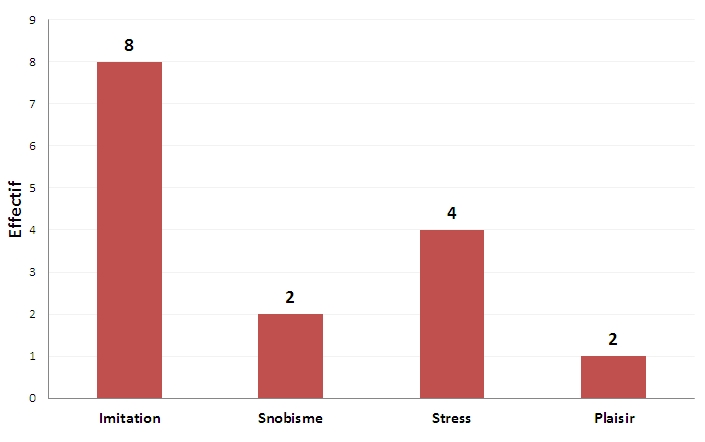
Facteurs initiateurs du tabagisme selon les répondants fumeurs (n=15)

L’âge moyen de début du tabagisme était de 21,5 ans avec des extrêmes de 12 à 30 ans. Le principal facteur initiateur du tabagisme était selon les répondants l'imitation (n=8), soit 53,3%; le stress y participait pour 26,7% (Figure 1). La durée moyenne de l'intoxication tabagique était de 24,7 ans avec des extrêmes de 10 à 40 ans.

Le type de tabac consommé était chez tous les fumeurs la cigarette. Le nombre quotidien de cigarettes fumées était de 7,2 avec des extrêmes de 2 à 15 cigarettes et un coût journalier variant de 350 à 3500 FCFA (0,53 à 5,3), le coût moyen étant de 1426 FCA (2,17).

Parmi les 15 fumeurs, 14 avaient essayé d'arrêter de fumer dans le passé dont 35,7% (n=5) au moins 3 fois. Les facteurs qui avaient motivé cet arrêt étaient une décision personnelle dans 60% des cas (n=9), un conseil médical dans 26,7% et la maladie dans 13,3%. La plus longue durée d'arrêt avait été de 5 ans et la plus courte de 15 jours avec une moyenne de 12,5 mois. Le score de Fagerström nous avait permis d'apprécier le degré de dépendance au tabac de nos fumeurs. La dépendance était faible (score de 3) pour 33,3 %, moyenne (score de 4 à 6) chez 53,3 % et forte (score de 7 à 10) dans 13,4 %.

Une consommation de cigarette à l'hôpital était observée chez 8 répondants (53,3%); cependant, aucun de ceux-ci ne fume à l'intérieur des bâtiments.

Tous les fumeurs souhaitent arrêter de fumer et 93,3% (n=14) d'entre eux pensaient avoir besoin d'un soutien pour les aider. Ce soutien espéré était psychologique dans 7,2% des cas (n=1), médicamenteux dans 21,4% (n=3), psychologique et médicamenteux dans 71,4% des cas (n=10).

### Caractéristiques des ex-fumeurs

**Figure 2:**
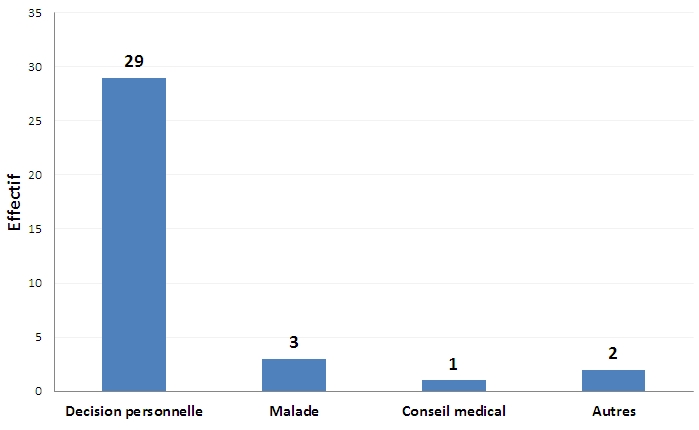
Les raisons d'arrêt du tabagisme données par les ex-fumeurs (n=35)

Trente-cinq sujets (13,4%) déclaraient être des ex-fumeurs. Ils avaient en moyenne arrêté de fumer depuis 13,5 ans avec des extrêmes de 1 à 36 ans. La décision personnelle était la principale raison d'arrêt du tabagisme (n=29), soit 82,9% (Figure 2).

### Connaissances du personnel

19,1% (n=53) personnel hospitalier était au courant de l'existence d'une loi anti tabac au Cameroun; 46,2% (n=128) ne savaient pas si cette loi existe et 34,7% (n=96) affirmaient qu'il n'existe pas de loi anti-tabac au Cameroun. Le personnel pensait être suffisamment informé des méfaits du tabagisme dans 79,7% (n=221).

### Attitudes du personnel face aux actions contre le tabac

La plupart des sujets (272/277) soit 97,9% répondaient que le personnel de santé doit montrer l'exemple à la population en ne fumant pas; six personnes (2,1%) pensaient le contraire.

**Figure 3:**
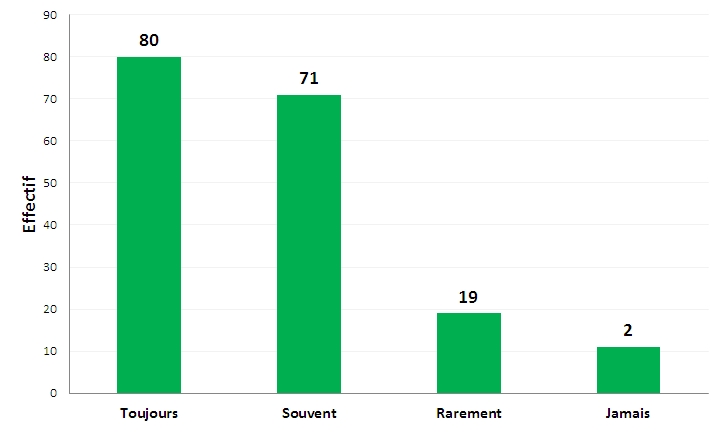
Demandez-vous à vos patients s'ils fument (n=181)

A la question de savoir s'ils demandaient à leurs patients s'ils fument, 80 sujets (44,2%) disaient toujours demander, tandis que 11 (6,1%) déclaraient ne jamais demander (Figure 3).

Parmi ceux qui posaient cette question aux patients, 56,4% (n=96) leurs conseillaient toujours, 27,6% (n=47) souvent, et 8,8% (n=15) rarement d'arrêter de fumer alors que 7,2% (n=12) ne donnaient jamais ce conseil à leurs patients.

### Opinion du personnel sur la lutte contre le tabac

Selon les répondants, les médias constituent le meilleur moyen de sensibilisation contre le tabac 36,8% (n=96) suivi des campagnes nationales anti-tabac (24,5%), des consultations d'aide au sevrage tabagique, de la publicité anti-tabac et des autres moyens. Ces autres moyens sont: la hausse du prix de la cigarette, l'arrêt de production du tabac, le porte à porte, la sensibilisation dans les établissements scolaires et dans les lieux de culte.

## Discussion

Le tabagisme chez le personnel de santé est important à étudier car, en plus du risque qu'il fait encourir aux fumeurs et aux non-fumeurs par le tabagisme passif, il altère l'image présentée au public par ce personnel. Par ailleurs, le tabagisme est une menace pour les performances de l'hôpital. Il peut être responsable d'accidents de travail, de maladies et de décès. L'hôpital doit être un lieu sans tabac afin que son personnel puisse servir de modèle pour les patients. En outre, un soignant fumeur ne contribuera pas de façon optimale à la lutte antitabac car il existe une liaison étroite entre le tabagisme du personnel hospitalier et son attitude par rapport à la lutte antitabac.

Ce travail a porté sur 402 employés de l'Hôpital Général de Douala parmi lesquels 277 nous avaient retourné leurs questionnaires. Les effectifs sont donc limités, en particulier pour le sous-groupe des fumeurs. Nous avons retrouvé 5,4% de fumeurs et 13,4% d'ex-fumeurs. Cette prévalence du tabagisme en milieu hospitalier est proche de celle retrouvée en milieu scolaire au Cameroun par Awono et al [2] qui reportaient 5,7% de fumeurs, inférieure à celle que l'OMS rapporte pour le Cameroun. Au Sénégal, Touré et al avaient trouvé 11,6% de fumeurs et 13,3% d'ex-fumeurs [3] et au Maroc, Alaoui et al notaient 14,9% de fumeurs et 7,6% d'ex-fumeurs [4]. En dehors du Maroc et du Sénégal, d'autres études faites en Afrique du Nord et en Europe et utilisant les mêmes critères d'inclusion que les nôtres avaient relevés des prévalences plus élevées [5–9]. Les administratifs et les agents de service fumaient plus que le personnel soignant. Ces résultats sont dénote un taux de tabagisme inférieur à celui d'autres travaux réalisés au Maroc [4], en Tunisie [7] et en France [8].

L’âge du début du tabagisme était en moyenne de 21,5 ans; des chiffres similaires étaient observés au Sénégal [3] et au Maroc [4]. Les facteurs initiateurs du tabagisme dans notre travail étaient dominés par l'imitation (53,3%) et le stress (26,7%). Ces facteurs sont variables selon les pays et les études; le plaisir est le principal facteur au Maroc [4,10,11] et au Sénégal, c'est le stress [3].

Dans notre travail, on ne notait pas de gros fumeurs (> 20 cigarettes/jour) tandis que Touré et al au Sénégal [3] trouvaient dans leur étude 31% de gros fumeurs. Toujours au Sénégal, Ndiaye et al [12] avaient trouvé plus de 50% de gros fumeurs au sein des fumeurs.

Tous nos fumeurs souhaitaient arrêter de fumer alors que Touré et al [3] rapportaient 86,2% de fumeurs manifestant ce désir. Ceci pourrait s'expliquer par le nombre plus élevés de fumeurs fortement dépendants observés dans ce travail (27,6%) par rapport à notre étude (13,4%). Les ex-fumeurs représentaient 13,4% de notre population d’étude. Cette prévalence est similaire à celle trouvée au Sénégal [4]. Contrairement aux résultats d'autres études [3, 4,12], dans notre travail, la décision personnelle était la principale motivation d'arrêt du tabagisme (82,9%).

Seulement 19,1% du personnel hospitalier était informé de l'existence d'une loi anti-tabac au Cameroun; ceci témoigne d'une sous information du public médical. Les pouvoirs publics devraient diffuser ce texte législatif à l'ensemble de la population camerounaise.

Ce travail nous permis d'avoir pour la première fois au Cameroun la prévalence du tabagisme chez le personnel de santé. Il s'agit d'une étude préliminaire et un travail à plus large échelle nous permettra d'avoir des données plus fiables sur le tabagisme en milieu hospitalier dans le pays.

## Conclusion

La lutte contre le tabagisme demeure une priorité pour l'OMS. Notre travail a trouvé une prévalence du tabagisme de 5,4%. Ce chiffre peut paraître faible par rapport à d'autre pays africains, mais compte tenu de montée du tabagisme chez les jeunes, les pouvoirs publics devraient intensifier la sensibilisation de la population et particulièrement le personnel hospitalier afin que l'hôpital devienne véritablement être un lieu sans tabac et que son personnel puisse servir de modèle pour les patients.
